# Supplementary material for: Microstructural Characterization and Magnetic, Dielectric, and Transport Properties of Hydrothermal La2FeCrO6 Double Perovskites
Source: Nanomaterials (Basel). 2023 Dec 13;13(24):3132. doi: 10.3390/nano13243132 (PMC10745924; doi:10.3390/nano13243132)
Supplement: Supplementary file 1 [file nanomaterials-13-03132-s001.zip › nanomaterials-2759167-supplementary.pdf]

Supplementary Materials

Microstructural Characterization and Magnetic, Dielectric, and Transport Properties of Hydrothermal La_2FeCrO_6 Double Perovskites

Section S1-Theoretical M_S value of the LFCO powders and their ASD content determination

To assess the theoretical M_S value of the LFCO powders based on an intuitive ionic model, one should take into account of the different oxide states of Fe and Cr ions and their relative amount ratios, as revealed by the XPS spectra. Here, the molar percentage ratios of $[Fe^{2+}]/[Fe^{3+}]$ and $[Cr^{3+}]/[Cr^{4+}]$ were 18%:82% and 74%:26%, respectively. The M_S value of the LFCO powders is evaluated based on the following Equations (S1-S2) and the electronic configurations of Fe^{2+} ($3d^6$, $\boldsymbol{t_{2g}^4 e_g^2}$; $\mu_{Fe^{2+}}$= 4.90 μ_B), Fe^{3+} ($3d^5$, $\boldsymbol{t_{2g}^3 e_g^2}$; $\mu_{Fe^{3+}}$= 5.92 μ_B), Cr^{3+} ($3d^3$, $\boldsymbol{t_{2g}^3 e_g^0}$; $\mu_{Cr^{3+}}$= 3.87 μ_B), and Cr^{4+} ions ($3d^2$, $\boldsymbol{t_{2g}^2 e_g^0}$; $\mu_{Cr^{4+}}$= 2.83 μ_B). In addition, the AFM coupling between the Fe and Cr ions is considered and the molar ratio of Fe:Cr is 1:1 at the B-sites. Thus,

$$< \boldsymbol{m_{Fe}} > = 4.90 \times 18\% + 5.92 \times 82\% = 5.74\ \mu_B/\text{f.u.} \qquad (S1)$$

$$< \boldsymbol{m_{Cr}} > = 3.87 \times 74\% + 2.83 \times 26\% = 3.60\ \mu_B/\text{f.u.} \qquad (S2)$$

$$M_S = < \boldsymbol{m_{Fe}} > - < \boldsymbol{m_{Cr}} > = 2.14\ \mu_B/\text{f.u} \qquad (S3)$$

In comparison with the above theoretical M_S (= 2.14 μ_B/f.u.), the experimental M_S = 0.31 μ_B/f.u. at 5 K, is much smaller due to the existence of large amounts of ASDs in the LFCO powders. The ASD content in the LFCO powders can be assessed by Equation (S4) [63]:

$$\boldsymbol{ASD} = \frac{\left(\boldsymbol{1}-\frac{\boldsymbol{M_s^{exp}}}{\boldsymbol{M_s^{cal}}}\right)}{\boldsymbol{2}} \qquad (S4)$$

where M_S^{cal} is the theoretical M_S value, and M_S^{exp} the experimental one. The ASD content was calculated to 42.8%, which reflected that the B-site ordering degree (η) of the LFCO powders was much smaller (14.4%), as described by Equation (S5) [64]:

$$\eta = 1 - 2*\text{ASD} \qquad (S5)$$

Section S2- Effective magnetic moments (μ_{eff}) and theoretical magnetic moment (μ_{cal})

By using the CW parameter C the effective magnetic moments (μ_{eff}) in the PM phase can be calculated as 6.64 μ_B/f.u. (@ 500 Oe) by the following Equation (S6) [65]:

$$\mu_{eff} = \sqrt{\frac{\boldsymbol{3k_B C}}{\boldsymbol{N_A \mu_B^2}}} = \boldsymbol{2.828\sqrt{C}} \qquad (S6)$$

As compared with the μ_{eff}, the theoretical magnetic moment, μ_{cal} (per formula unit) of the $La_2\boldsymbol{Fe_{0.18}^{2+}Fe_{0.82}^{3+}Cr_{0.74}^{3+}Cr_{0.26}^{4+}}O_6$ is determined to be 6.80 μ_B/f.u. by using Equation (S7) [66]:

$$\boldsymbol{\mu_{cal}} = \sqrt{\boldsymbol{0.18}\times\boldsymbol{\mu_{Fe^{2+}}^2} + \boldsymbol{0.82}\times\boldsymbol{\mu_{Fe^{3+}}^2} + \boldsymbol{0.74}\times\boldsymbol{\mu_{Cr^{3+}}^2} + \boldsymbol{0.26}\times\boldsymbol{\mu_{Cr^{4+}}^2}} \qquad (S7)$$

where the effective magnetic moments for the Fe^{2+} ($\boldsymbol{\mu_{Fe^{2+}}}$= 4.90 μ_B), Fe^{3+} ($\boldsymbol{\mu_{Fe^{3+}}}$= 5.92 μ_B), Cr^{3+} ($\mu_{Cr^{3+}}$= 3.87 μ_B), and Cr^{4+} ($\mu_{Cr^{4+}}$= 2.83 μ_B) ions are used [67].

Table S1 Refined crystal structural parameters (space group, unit cell parameters, average bond lengths and bond angles), structural tolerance factor (t), reliability factors, and average crystallite size as well as the magnetic data (M_S, M_r, H_C, T_N, irreversibility temperature T_{irr}, Curie-Weiss constant C and paramagnetic Curie-Weiss temperature θ_p, effective paramagnetic moment, μ_{eff} calculated from Curie-Weiss constant C, and theoretically calculated magnetic moment, μ_{cal} of the hydrothermal LFCO oxides.

Materials parameters	Measured data for the hydrothermal LFCO oxides
Space group	*Pnma*
Unit cell parameters	a = 5.5293(6) Å, b = 7.8192(0) Å, c = 5.5408(9) Å, $\alpha = \beta = \gamma$ = 90°, V = 239.56(7) Å^3
Cr-O bond length (Å)	
Cr_1-O_1	1.9928(2)
Cr_1-O_2	1.9918(1)
Cr_1-O	1.9923(1)
Fe-O bond length (Å)	
Fe_1-O_1	1.9928(2)
Fe_1-O_2	1.9918(1)
Fe_1-O	1.9923(1)
<∠Fe-O_i-Cr> bond angle (°)	158.559(3)
∠Fe_1-O_1-Cr_1	157.529(2)
∠Fe_1-O_2-Cr_1	158.528(3)
Structural tolerance factor (t)	0.961 calculated by $t = \frac{r_{La^{3+}} + r_{O^{2-}}}{\sqrt{2}\left(\frac{r_{Cr^{3+}} + r_{Fe^{3+}}}{2} + r_{O^{2-}}\right)}$
Reliability factors	R_{wp} = 6.03%; R_p = 5.98%; χ^2 = 2.32
Average crystallite size (nm)	41 nm calculated by Scherrer formula (Eq. (1))

Magnetic data	2 K			T_N (K)	T_B (K)	T_C (K)	θ_P (K)	T_{irr} (K)	C @ 500 Oe (emu·K/mol)	μ_{eff} (μ_B/f.u.)	μ_{cal} (μ_B/f.u.)
	M_S (μ_B/f.u.)	M_r (μ_B/f.u.)	H_c (kOe)								
	0.31	0.021	8.0	10	113	200	-441	295	5.51	6.64	6.80

Table S2 Species, peak positions and percentage molar ratios obtained from the peak fittings of Fe 2$p_{3/2}$, Cr 2$p_{3/2}$and O 1s XPS spectra of the hydrothermal LFCO powders and the effective oxidation states of the Fe and Cr elements.

XPS results		Hydrothermal LFCO powders		
XPS spectra	Fe 2$p_{3/2}$	Species	Position (eV)	Percentage molar ratio
		Fe^{2+}	710.15	$[Fe^{2+}]/[Fe^{3+}]$ = 18%:82%
		Fe^{3+}	711.41	
	Cr 2$p_{3/2}$	Species	Position	Percentage molar ratio
		Cr^{3+}	576.25	$[Cr^{3+}]:[Cr^{4+}]$ = 74%:26%
		Cr^{4+}	578.90	
	O 1s	Species	Position	Percentage molar ratio
		O_α	529.42	$[O_\alpha]:[O_\beta]$ = 46%: 54%
		O_β	531.70	
Effective oxide states of Fe and Cr elements				Fe element: + 2.82<br>Cr element: + 3.26

References

63. Pal, S.; Govinda, S.; Goyal, M.; Mukherjee, S.; Pal, B.; Saha, R.; Sundaresan, A.; Jana, S.; Karis, O.; Freeland, J.W.; et al. Effect of anti-site disorder on magnetism in La_2NiMnO_6. *Phys. Rev. B* **2018**, *97*, 165137.
64. Moritomo, Y.; Shimamoto, N.; Xu, S.; Machida, A.; Nishibori, E.; Takata, M.; Sakata, M.; Nakamura, A. Effects of B-site disorder in Sr_2FeMoO_6 with double perovskite structure. *J. Appl. Phys.* **2001**, *40*, L672–L674.
65. Rogado, N.S.; Li, J.; Sleight, A.W.; Subramanian, M.A. Magnetocapacitance and magnetoresistance near room temperature in a ferromagnetic semiconductor: La_2NiMnO_6. *Adv. Mater.* **2005**, *17*, 2225–2227.
66. Taguchi, H. Relationship between crystal structure and electrical properties of $Nd(Cr_{1-x}Fe_x)O_3$. *J. Solid State Chem.* **1997**, *131*, 108–114.
67. Buschow, K.H.J.; Boer, F.R. *Physics of Magnetism and Magnetic Materials*; Springer: Berlin/Heidelberg, Germany, 2003.
